# Comparison of Envelope-Related Genes in Unicellular and Filamentous Cyanobacteria

**DOI:** 10.1155/2007/25751

**Published:** 2007-08-06

**Authors:** Yu Yang, Song Qin, Fangqing Zhao, Xiaoyuan Chi, Xiaowen Zhang

**Affiliations:** ^1^ Institute of Oceanology, Chinese Academy of Sciences, Qingdao 266071, China; ^2^ Graduate University, Chinese Academy of Sciences, Beijing 100049, China

## Abstract

To elucidate the evolution of cyanobacterial envelopes and the relation between gene content and environmental adaptation, cell envelope structures and components of unicellular and filamentous cyanobacteria were analyzed in comparative genomics. Hundreds of envelope biogenesis genes were divided into 5 major groups and annotated according to their conserved domains and phylogenetic profiles. Compared to unicellular species, the gene numbers of filamentous cyanobacteria expanded due to genome enlargement effect, but only few gene families amplified disproportionately, such as those encoding waaG and glycosyl transferase 2. Comparison of envelope genes among various species suggested that the significant variance of certain cyanobacterial envelope biogenesis genes should be the response to their environmental adaptation, which might be also related to the emergence of filamentous shapes with some new functions.

## 1. INTRODUCTION

As the oldest oxygenic phototrophs on the earth, cyanobacteria originated 2.8∼3.5 billion years ago [1]. Cyanobacteria are usually considered gram negative in traditional classification of prokaryotic envelopes [2], for the existence of outer membrane and lack of teichoic acid in cell walls. However, many unusual features exist in their envelopes. Cyanobacteria have a thick (15∼35 nm or more) and high cross-linking peptidoglycan layer, similar to gram-positive bacteria [3]. Some rare composition of gram-negative walls, such as carotenoid [4] and β-hydroxypalmitic acid [5], has been found from in lipopolysaccharide (LPS) of cyanobacteria. The archaic organisms contain cellulose indicative of vascular plants [6].

Phylum cyanobacteria has been well diverged in evolution. Some cyanobacteria have been evolved in a multicellular filamentous form, while others remained unicellular. Filamentous cyanobacteria are the oldest known multicellular organisms [7], and the divergence of cyanobacteria is a landmark in biological evolution. Transition from unicellular to filamentous cyanobacteria was a significant evolutionary event, as the organisms were equipped with an advantageous interior nutrition system able to interact with ambient factors [8].

The rise of genomics greatly promoted biological research, of which comparative genomics became an effective tool to explore different species. So far, 25 cyanobacterial genomes, both unicellular and filamentous, have been sequenced, ranging from 1.6 to 9.1 Mb [9]. However, a large difference exists in cell envelope between unicellular and filamentous species. At present, few comparative analyses have been made concerning the structure and function of cell envelopes of both. Therefore, to understand the diversity in cyanobacterial envelope, comparative genomic analysis is conducted in this paper by comparing envelope biogenetic genes between unicellular and filamentous species. As each of them occupies own ecological niche, cyanobacterial genome, the envelope structure, and environment adaptability were associated for inferring multicellular selection of cyanobacteria.

## 2. MATERIALS AND METHODS

### 2.1. The information management system

At the time of this study, 25 sequenced cyanobacterial genomes, including 21 unicellular and 4 filamentous were available for public online access into the Integrated Microbial Genomes (IMG) system provided by Joint Genome Institute (JGI) (http://img.jgi.doe.gov/cgi-bin/pub/main.cgi) [10]. Unicellular *Prochlorococcus marinus* MED4 and *Synechocystis* sp. PCC 6803, and filamentous *Trichodesmium erythraeum* IMS101 and *Anabaena* sp. PCC 7120 (also called *Nostoc* sp. PCC 7120) were chosen for this research. In each species, over 60% of genes have been already included into the database of Clusters of Orthologous Groups (COGs) [11] based on orthology concept [12]. In a COG under the directory of “Cell wall/membrane/envelope biogenesis,” gene sequences in FASTA amino acid format were selected, exported, and downloaded in November, 2006 (as IMG version often updates, the data may change).

### 2.2. Gene retrieval and annotation

Quite a number of genes directly available online have only accession or gene ID, but complete description. So it was hard to know their roles in cyanobacterial envelope biogenesis. What we tried to solve the problem was to online-use software InterProScan from the EMBL of European Bioinformatics Institute (EBI) (http://www.ebi.ac.uk/InterProScan) [13]. Unfortunately, this action alone could not provide enough information, such as the family to which the gene belongs and the impact by envelope biogenesis. Therefore, two online tools in NCBI, protein-protein BLAST (blastp), (http://www.ncbi.nlm.nih.gov/BLAST) [14] and reverse position specific BLAST (RPS-BLAST) (http://www.ncbi.nlm.nih.gov/Structure/cdd/wrpsb.cgi) [15], were also used as assistants.

Putative conserved domains of the genes (without detailed description) were detected; and the genes were aligned up with other known genes, commonly with score > 80 bits and expect < 1e-10 at least. Finally, present references to the roles of particular domains or gene families involved in bacterial envelope biogenesis were combined; the unclear genes would be retrieved and annotated.

### 2.3. Sequence alignment and phylogenetic analysis

The sequences with similar domains were input and completely aligned using ClustalX 1.8. The produced files with “^*^.aln” extension were opened by BioEdit at the option of “Graphic View.” The same or similar residues were highlighted in black or dark shade. In this paper, only the most conserved area of gene sequences is shown in figures.

In addition to *Trichodesmium erythraeum* IMS101 and *Anabaena* sp. PCC 7120, 15 FAS1-containing genes from other cyanobacteria, archaebacteria, eubacteria, yeast, filamentous fungi, and high plants were gained from NCBI. Sequence alignments of genes predicted for the same families were used as an input file for MEGA3 program [16]. Phylogenetic tree was built via the Neighbor-Joining (NJ) method in evaluation with 1000 rounds of bootstrapping test [17, 18].

## 3. RESULTS

One hundred envelope biogenesis genes were obtained from *Prochlorococcus marinus* MED4, 186 from *Synechocystis* sp. PCC 6803, 266 from *Trichodesmium erythraeum* IMS101, and 294 from *Anabaena* sp. PCC 7120, which are shown in the “total” column in Table 1. Known constituents of cyanobacterial cell walls, including peptidoglycan, lipopolysaccharide (LPS), exopolysaccharide (EPS), outer membrane protein, and so on, were respectively synthesized under the control of different genes. Thus we might as well divide above 846 envelope biogenesis genes into 5 major types: peptidoglycan biosynthesis-related (PBR) genes, lipopolysaccharide biosynthesis-related (LBR) genes, exopolysaccharide biosynthesis-related (EBR) genes, outer membrane proteins (OMP) coding genes, and other unknown (OU) genes. The OU ones were loaded from the COG “Cell wall/membrane/envelope biogenesis;” but not enough information was available to annotate them using the methods mentioned in the section “Gene retrieval and annotation.”

Table 1 shows the absolute and relative amounts of classified genes from unicellular and filamentous species. The appearance of filament naturally resulted in the enlargement of genome sizes and the addition of gene numbers; however, the percentage of each type of “total” varied, too. Therefore, the percentage of EBR increased in filamentous species (EBR percentage of *Trichodesmium erythraeum* IMS101 and *Anabaena* sp. PCC 7120 was 18.0% and 21.2% respectively, compared with 15.0% of *Prochlorococcus marinus* MED4 and 15.1% of *Synechocystis* sp. PCC 6803). The percentage of other types changed simultaneously, which were discussed in detail in Section 4.

### 3.1. Percentage variation of peptidoglycan biosynthesis-related (PBR) genes

Being an important component of cyanobacterial envelope, peptidoglycan forms a covalently closed and net-like layer, for protecting cells against detrimental environmental influences, maintaining a high internal osmotic pressure, and serving as a barrier to transenvelope transport sometimes [19]. As the amount of envelope biogenesis gene from *Prochlorococcus marinus* MED4 to *Anabaena* sp. PCC 7120 increased, this increase was exclusively reflected on one gene family, which encodes class A high-molecular-weight penicillin binding proteins [20]. However, the percentage of PBR decreased instead. In filamentous cyanobacteria, envelope components (besides peptidoglycan) and structures could also protect the cells, such as exopolysaccharide and filamentous sheaths; so relatively fewer peptidoglycan genes were expressed.

### 3.2. Uneven increase of LBR genes in filamentous cyanobacteria

LPS also has a function of the protection, so the percentage of LBR genes of “total” decreased from unicellular to filamentous cyanobacteria, which is like PBR genes. This course is clearly expressed among *Prochlorococcus marinus* MED4, *Synechocystis* sp. PCC 6803, and *Trichodesmium erythraeum* IMS101. However, *Anabaena* sp. PCC 7120 did not obey the “trend.” It expressed relatively more LBR genes than that of *Trichodesmium erythraeum* IMS101, which is probable due to differentiation of some cells into heterocysts, forming special N_2_-fixing cells within O_2_-producing filamentous cyanobacteria [20, 21]. For nitrogen fixing, the heterocysts need extracellular LPS layers to protect oxygen invasion [22].

**Figure 1:**
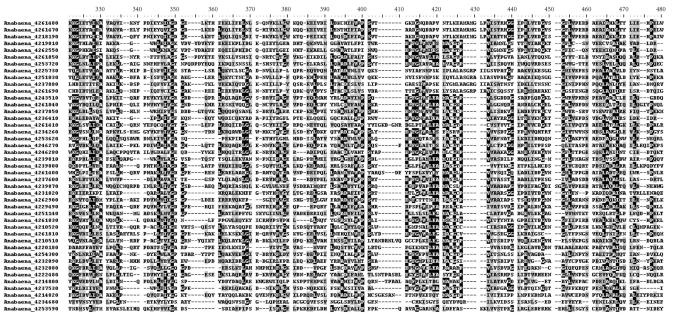
Multiple sequence alignments of the 43 *waaG* homologous genes in *Anabaena* sp. PCC 7120. Only most conserved areas were shown. The number following the genus name was the gene accession in IMG database. NCBI accessions and other information of genes were provided in *Table 2*.

In terms of absolute amounts, *Anabaena* sp. PCC 7120 had most of the LBR genes. Interesting is that most increased genes had the common conserved domain waaG (formerly RfaG). There were 43 waaG-containing genes found in *Anabaena* sp. PCC 7120 (while only 5 in *Prochlorococcus marinus* MED4, 17 in *Synechocystis* sp. PCC 6803, and 24 in *Trichodesmium erythraeum* IMS101). The 43 genes and their multiple alignments in similar domain were shown in Table 2 and Figure 1, about 20 residues out of the 43 sequences were in common (black shading areas). These residues may have formed typical spatial structures that could be defined as active sites of waaG domain.

The *waa* family includes many members, such as *waaP*, *waaY*, *waaA*, *waaT*, *waaO*, *waaQ*, *waaA*, and *waaC*, and helps synthesize the LPS core oligosaccharide. At present, we only knew that the *waaG* product is a glucosyltransferase, and its mutation can truncated LPS at the phosphorylation sites and destabilized the outer membrane [23]. Probably, *waaG* can provide a selective advantage to *Anabaena* sp. PCC 7120.

### 3.3. Analysis of EBR

**Figure 2:**
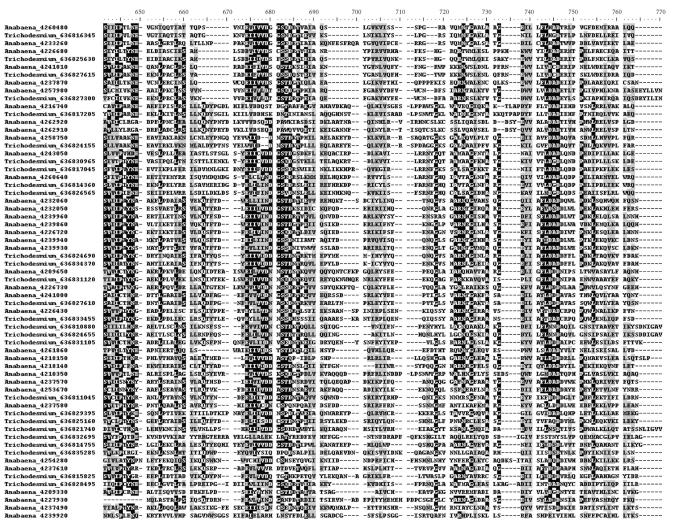
Multiple sequence alignments of homologous genes encoding glycosyl transferase 2 (GT2) domains in *Trichodesmium erythraeum* IMS101 (27 genes) and *Anabaena* sp. PCC 7120 (36 genes). Only most conserved areas were shown. The number following the genus name was the gene accession in IMG database. NCBI accession and other information of genes were provided in Tables 3 and 4.

During the progress from unicellular to filamentous cyanobacteria, the percentage of EBR genes increased clearly but unevenly in some particular genes. Most extra genes of filamentous species belonged to the family encoding glycosyl transferase 2 that involved in many metabolic processes, mainly in the cellulose biosynthesis [24]. The common conserved domain Glycos_transf_2 was detected for 36 times in *Anabaena* sp. PCC 7120, and 27 in *Trichodesmium erythraeum* IMS101, as shown in Tables 3 and 4 and Figure 2, whereas it was only 8 times in *Prochlorococcus marinus* MED4 and 14 times in *Synechocystis* sp. PCC 6803. It is believed that certain member in the family glycosyl transferase 2 was a key enzyme synthesizing cellulose in filamentous cyanobacteria.

**Figure 3:**
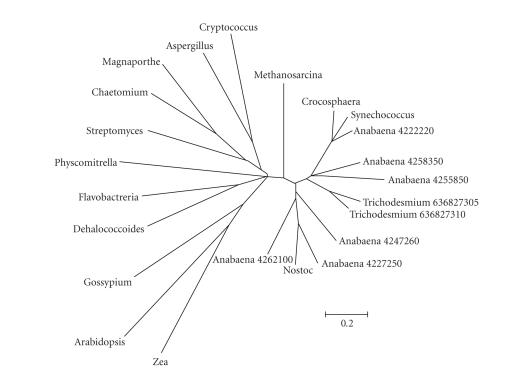
The phylogenetic tree of genes containing FAS1 domain in 17 species. Besides *Trichodesmium erythraeum* IMS101 and *Anabaena* sp. PCC 7120, other 15 species were from cyanobacteria, archaebacteria, eubacteria, actinomycetes, yeast, filamentous fungi, and vascular plants. To keep the figure clear and direct, the species were written in their genus name for short. The detailed information was described in Section 3 and *Table 5*.

Fasciclin-like (FAS1) family has been identified as hemicellulose synthase in fungi and high plants [25], and it was involved in the secondary wall biosynthesis [26]. Homologues of this conserved domain, closely relative to the formation of filaments and extracellular polysaccharides, has been found in archaebacteria, eubacteria, actinomycetes, yeast, filamentous fungi, and vascular plants. It was found that 2 genes in *Trichodesmium erythraeum* IMS-101 and 6 in *Anabaena* sp. PCC 7120 contained the domain. Representative FAS1-containing genes were found from NCBI, including *Synechococcus*, *Crocosphaera*, *Nostoc*, *Methanosarcina*, *Dehalococcoides*, *Aspergillus*, *Cryptococcus*, *Flavobacteria*, *Physcomitrella*, *Streptomyces*, *Chaetomium*, *Magnaapothe*, *Arabidopsis*, *Gossypium*, and *Zea*, as shown in Table 5. Phylogenetic tree of all 23 FAS1-containing genes in many species was constructed (See Figure 3). It is clear that genes in *Trichodesmium erythraeum* IMS101 and *Anabaena* sp. PCC 7120 were distant from other cyanobacteria (*Synechocystis*, *Synechococcus*, *Crocosphaera*, and *Nostoc*); and all the cyanobacterial genes were separated from fungi and plants. The FAS1-containing genes were paralogous in the Phylum Cyanobacteria.

## 4. DISCUSSION

### 4.1. General descriptions of 5 types of genes

In Table 1, remarkable changes could be seen from top to bottom lines, especially in columns of PBR, EBR, and OMP, which should be easily understood: to adopt better external environment and improve own nutrition status, cyanobacterial envelopes have to be modified. Adding outer membrane proteins could be a choice, as happened in *Synechocystis* sp. PCC 6803. From unicellular to filamentous cyanobacteria, the number of envelope biogenesis gene has increased. However, the increase was uneven, and gene duplication focused on in very few families. It is shown that in the evolution, only few families of genes expressed excessively, and they could be involved in generating novel structures and functions.

### 4.2. Role of waaG in filamentous cyanobacterial regulation

LPS is a characteristic component of gram-negative bacteria, which shows architecture of 3 covalently linked domains, namely hydrophobic lipid A, core oligosaccharide, and hydrophilic O-antigen [27]. In structural feature, the region of phosphorylated core oligosaccharide can be subdivided into inner and outer cores [28]. During LPS biosynthesis, *waaG* produces transferases, a glucosyl group from D-glucose I (Glc I) I to L-glycero-D-manno-heptose II (Hep II). The mutation of *waaG* destabilizes the LPS layer by interfering with core phosphorylation [23]; and the stability of LPS layer is necessary to the stabilization of heterocysts' external layers [22]. Unlike marine filamentous *Trichodesmium erythraeum* IMS101, *Anabaena* sp. PCC 7120 usually lives in freshwater or wetland, which is considered as a less stable environment than marine ecosystem, with drastic changes of temperature and light, abundant but inconstant nutrient resources and more potential hazards. *Anabaena* sp. PCC 7120 is also able to produce heterocysts to fix N_2_ and actively adapt environment, making itself more mutable than in the ocean environment. Over-expression of *waaG* homologous genes would help stabilize the heterocysts, and improve the N_2_-fixing in *Anabaena* sp. PCC 7120.

### 4.3. Relation between EBR and cyanobacterial evolution

Cyanobacterial filaments were made up of mainly diverse polysaccharide molecules, containing cellulose and matrix polysaccharide. Most of the genes are from the glycosyl transferase 2 (GT2) family. In model plant *Arabidopsis thaliana*, over 10 members of the family catalyze glucan-chain elongation in cellulose, and they belong to the group of genes encoding catalytic subunit of cellulose synthase (CESA) [29]. Since cellulose and other EPS were also the main components of cyanobacterial filamentous sheath, the GT2 family may play a vital role in the formation of filaments. In the meanwhile, these results could further prove that the cellulose produced by cyanobacteria is, at least one of, the earliest origins of the most abundant biopolymer on the earth today [30].

At present, a little is known about the matrix polysaccharide (hemicellulose, pectin, and so on) in cyanobacteria. Surprisingly, several matrix polysaccharide biogenesis genes or their homologues were discovered in this study. The phylogenetic tree (see Figure 3) shows that the genes of fasciclin-like (FAS1) family are duplicated in evolution among different cyanobacteria, suggesting that the FAS1 family occurred after the branch point where cyanobacteria separated from other archaic species but before the divergence of different cyanobacteria. The family is very rare in oceanic unicellular cyanobacteria, but in filamentous *Anabaena*, *Nostoc*, and *Trichodesmium*, it cannot be neglected. Large difference in content of the family between unicellular and filamentous cyanobacteria implied the family's contribution to filament formation, which provides us a clue to understanding the evolution of cyanobacteria.

### 4.4. Species selection and gene classification

Contrast to *Prochlorococcus marinus* MED4, *Trichodesmium erythraeum* IMS101, and *Anabaena* sp. PCC 7120, the selection of *Synechocystis* sp. PCC 6803 is more or less special. Although *Synechocystis* sp. PCC 6803 is usually unicellular in entire lifetime, it can hardly be recognized as a “pure” unicellular cyanobacterium. *Synechocystis* sp. PCC 6803 can frequently congregate in dimer or in a loose group. The group members must communicate with each other in special mechanism with similar actions to multicellular filaments. Strong light or other external factors can stimulate numerous single cells to arrange in filamentous shape, showing semi-filamentous feature. That was why *Synechocystis* sp. PCC 6803 was chosen for this study as it has clear transitional character. Selection of and comparison among the 4 cyanobacteria with own particular shape and status should be more persuasive on the issue of this paper.

In addition to major components of typical gram-negative bacteria, the existence of EPS (mainly refers to cellulose and hemicellulose) in cyanobacteria is significant. Therefore, peptidoglycan, LPS, EPS, and outer membrane proteins become 4 major components of cyanobacterial envelopes. Over 93% of biogenesis genes of each cyanobacterial envelope were placed in correct place, leaving only <7% of other unknown genes, showing that the classification is scientifically acceptable and also practical. However, problem still remains as it is difficult to eliminate error or misplacement until all cyanobacterial genes are correctly annotated. For instance, some LBR coding proteins were localized in the outer membrane; so these LBR genes can also be considered as OMP genes. Therefore, the genes of the OMP defined in this paper represented mostly those genes whose expressing products are located in outer membrane and carry out functions other than the biosynthesis by peptidoglycan, LPS, and EPS.

Moreover, previous reports believed that cyanobacterial cell wall did not contain teichoic acid [3], but the gene *alr4011* in *Anabaena* sp. PCC 7120 put the issue in argument. The amino acid sequence of *alr4011* has a conserved domain DltE that is a short-chain dehydrogenase involved in the teichoic acid synthesis [31]; and *alr4011* showed great similarity to the gene *dltE* in gram-positive *Bacillus subtilis* (146 bits [Expect = 7e-34]). No DltE-containing gene was found in *Prochlorococcus marinus* MED4, *Synechocystis* sp. PCC 6803, or *Trichodesmium erythraeum* IMS101. A possible explanation is that *alr4011* was transferred horizontally from gram-positive bacteria, or that the gene was regulated via a special pathway to produce another envelope constituent but teichoic acid. Whether teichoic acid exists in cyanobacterial envelopes is currently an open question that needs further research and experiment.

## Figures and Tables

**Table 1:** Absolute and relative numbers of envelope related genes in four cyanobacteria. PBR, LBR, EBR, OMP, and OU represent peptidoglycan biosynthesis-related, lipopolysaccharide biosynthesis-related, exopolysaccharide biosynthesis-related, outer membrane proteins coding, and other unknown genes, respectively. The data in the brackets were the percentage of each group within the total envelope-related genes.

Species	Total	PBR	LBR	EBR	OMP	OU
*Prochlorococcus marinus* MED4	100	29 (29.0%)	40 (40.0%)	14 (15.0%)	16 (15.0%)	2 (2.0%)
*Synechocystis* sp. PCC 6803	186	37 (19.9%)	73 (39.2%)	28 (15.1%)	40 (21.5%)	8 (4.3%)
*Trichodesmium erythraeum* IMS101	266	47 (17.7%)	90 (33.1%)	48 (18. 0%)	63 (23.7%)	18 (6.8%)
*Anabaena* sp. PCC 7120	294	48 (16.3%)	113 (38.4%)	61 (20.7%)	60 (20.4%)	12 (4.1%)

**Table 2:** *waaG* homologous genes in *Anabaena* sp. PCC 7120. Information of the 43 genes was provided.

NCBI accession	IMG accession	Locus Tag	Product	Position in Genome
NP_484203	4210510	Alr0159	Alr0159 protein	163382–164575
NP_484204	4210520	All0160	All0160 protein	164558–165712
NP_484626	4214800	Alr0582	Alr0582 protein	676349–677545
NP_484628	4214820	Alr0584	Alr0584 protein	679928–681130
NP_484962	4218190	All0919	All0919 protein	1063224–1064513
NP_485043	4219010	Alr1000	Alr1000 protein	1171949–1173031
NP_485160	4220180	Alr1117	Alr1117 protein	1308038–1309267
NP_485388	4222480	All1345	All1345 protein	1596626–1597858
NP_485708	4225730	Alr1668	Alr1668 protein	1990621–1991904
NP_486077	4229490	All2037	All2037 protein	2435914–2437014
NP_486305	4231820	SqdX	Sulfolipid sulfoquinovosyldiacylglycerol biosynthesis protein	2725143–2726279
NP_486331	4232080	All2291	Glycosyltransferase	2760187–2761173
NP_486332	4232090	All2292	All2292 protein	2761170–2762348
NP_486547	4234260	All2507	All2507 protein	3008236–3009423
NP_486589	4234680	All2549	All2549 protein	3051362–3052363
NP_486760	4236410	All2720	All2720 protein	3315625–3316713
NP_486872	4237530	Alr2832	Alr2832 protein	3448705–3449793
NP_486879	4237600	Alr2839	Glycosyltransferase	3459432–3460577
NP_486904	4237850	Alr2864	Alr2864 protein	3488401–3489579
NP_486907	4237880	Alr2867	Alr2867 protein	3491419–3492636
NP_487097	4239800	Alr3057	Alr3057 protein	3703378–3704592
NP_487098	4239810	Alr3058	Alr3058 protein	3704628–3705854
NP_487104	4239870	Alr3064	Alr3064 protein	3712759–3714171
NP_487465	4243510	Alr3425	Alr3425 protein	4133859–4135025
NP_487738	4246270	HepB	Heterocyst envelope polysaccharide synthesis protein	4465828–4466997
NP_487739	4246280	Alr3699	Alr3699 protein	4467059–4468207
NP_488208	4251030	Alr4168	Alr4168 protein	5015231–5016502
NP_488218	4251140	Alr4178	Alr4178 protein	5025948–5027096
NP_488463	4253590	All4423	All4423 protein	5300887–5302026
NP_488466	4253620	All4426	All4426 protein	5304172–5305425
NP_488476	4253720	All4436	All4436 protein	5320348–5321526
NP_488534	4254300	Alr4494	Mannosyltransferase	5380744–5381811
NP_489234	4261400	All5194	Glycosyltransferase	6192395–6193555
NP_489235	4261410	All5195	Glycosyltransferase	6193736–6194992
NP_489241	4261470	Alr5201	Glycosyltransferase	6201983–6203275
NP_489242	4261480	Alr5202	Glycosyltransferase	6203285–6204574
NP_489263	4261690	Alr5223	Glycosyltransferase	6236642–6237991
NP_489275	4261810	Alr5235	Alr5235 protein	6247505–6248551
NP_489277	4261830	Alr5237	Alr5237 protein	6249905–6251158
NP_489278	4261840	Alr5238	Glycosyltransferase	6251167–6252315
NP_489279	4261850	Alr5239	Alr5239 protein	6252417–6253586
NP_489347	4262550	Alr5307	Alr5307 protein	6328387–6329490
NP_489381	4262900	All5341	All5341 protein	6373814–6375079

**Table 3:** Genes encoding GT2 domain in *Anabaena* sp. PCC 7120. Information of the 36 genes was provided.

NCBI Accession	IMG Accession	Locus Tag	Product	Position in Genome
NP_484086	4209330	all0042	All0042 protein	44511–45458
NP_484118	4209650	alr0074	Alr0074 protein	78171–79187
NP_484187	4210350	all0143	All0143 protein	148503–149681
NP_484819	4216740	alr0776	Alr0776 protein	899704–900894
NP_484957	4218140	all0914	All0914 protein	1057871–1058884
NP_484958	4218150	all0915	All0915 protein	1058947–1059852
NP_485777	4226430	all1737	All1737 protein	2088106–2089074
NP_485802	4226680	all1762	All1762 protein	2117622–2118518
NP_485806	4226720	all1766	All1766 protein	2121006–2122007
NP_485807	4226730	all1767	All1767 protein	2122000–2123007
NP_485926	4227930	all1886	All1886 protein	2252568–2253362
NP_486328	4232050	all2288	Glucosyltransferase	2756810–2757841
NP_486329	4232060	all2289	Glucosyltransferase	2757927–2758916
NP_486448	4233260	alr2408	Alr2408 protein	2888194–2888949
NP_486868	4237490	alr2828	Alr2828 protein	3444428–3445441
NP_486876	4237570	alr2836	Putative glycosyl transferase	3456248–3457216
NP_486877	4237580	alr2837	Glycosyltransferase	3457336–3458310
NP_486880	4237610	alr2840	Glycosyltransferase	3460577–3461524
NP_486906	4237870	alr2866	Glycosyltransferase	3490561–3491400
NP_487103	4239860	alr3063	Alr3063 protein	3711770–3712762
NP_487109	4239920	alr3069	Alr3069 protein	3718782–3719963
NP_487110	4239930	alr3070	Alr3070 protein	3719986–3720942
NP_487111	4239940	alr3071	Alr3071 protein	3720982–3721938
NP_487113	4239960	alr3073	Alr3073 protein	3723391–3724365
NP_487216	4241000	alr3176	Alr3176 protein	3844812–3845753
NP_487217	4241010	alr3177	Alr3177 protein	3845774–3846715
NP_487420	4243050	alr3380	Dolichol-phosphate mannosyltransferase	4091498–4092511
NP_488471	4253670	all4431	Glycosyl transferase	5310064–5311017
NP_488532	4254280	alr4492	Alr4492 protein	5378788–5379816
NP_488897	4257980	all4857	All4857 protein	5785088–5786275
NP_488973	4258750	all4933	All4933 protein	5886142–5887548
NP_489142	4260480	all5102	All5102 protein	6079688–6080410
NP_489158	4260640	all5118	All5118 protein	6114366–6115355
NP_489280	4261860	alr5240	Glycosyltransferase	6253630–6254397
NP_489382	4262910	all5342	All5342 protein	6375223–6376452
NP_489383	4262920	all5343	All5343 protein	6376587–6377849

**Table 4:** Genes encoding GT2 domain in *Trichodesmium erythraeum* IMS101. Information of the 27 genes was provided.

NCBI accession	IMG accession	Locus Tag	Product	Position in Genome
YP_720085	636810880	Tery_0115	Glycosyl transferase, family 2	155085–157763
YP_720116	636811045	Tery_0148	Glycosyl transferase, family 2	217777–218829
YP_720694	636814360	Tery_0804	Glycosyl transferase, family 2	1279953–1280891
YP_720758	636814755	Tery_0883	Glycosyl transferase, family 2	1403156–1404088
YP_720935	636815825	Tery_1097	Glycosyl transferase, family 2	1725763–1726743
YP_721031	636816345	Tery_1201	Glycosyl transferase, family 2	1875929–1876612
YP_721128	636817045	Tery_1340	Glycosyl transferase, family 2	2040749–2041705
YP_721156	636817205	Tery_1372	Glycosyl transferase, family 2	2104828–2106021
YP_721969	636821740	Tery_2268	Glycosyl transferase, family 2	3529656–3534458
YP_722405	636824155	Tery_2749	Glycosyl transferase, family 2	4257314–4258822
YP_722496	636824655	Tery_2849	Glycosyl transferase, family 2	4430305–4432779
YP_722503	636824690	Tery_2856	Glycosyl transferase, family 2	4447185–4448186
YP_722586	636825160	Tery_2950	Glycosyl transferase, family 2	4584744–4585874
YP_722664	636825630	Tery_3040	Glycosyl transferase, family 2	4692416–4693294
YP_722816	636826565	Tery_3225	Glycosyl transferase, family 2	4937986–4938924
YP_722946	636827300	Tery_3371	Glycosyl transferase, family 2	5168831–5170021
YP_722999	636827610	Tery_3433	Glycosyl transferase, family 2	5251339–5252268
YP_723000	636827615	Tery_3434	Glycosyl transferase, family 2	5252486–5253415
YP_723155	636828495	Tery_3609	Glycosyl transferase, family 2	5550523–5551482
YP_723304	636829395	Tery_3784	Dolichyl-phosphate beta-D-mannosyltransferase	5816905–5817705
YP_723576	636830965	Tery_4095	Glycosyl transferase, family 2	6315001–6316008
YP_723603	636831105	Tery_4122	Glycosyl transferase, family 2	6360766–6361701
YP_723606	636831120	Tery_4125	Glycosyl transferase, family 2	6363768–6364736
YP_723897	636832695	Tery_4437	Glycosyl transferase, family 2	6839236–6842421
YP_724037	636833455	Tery_4588	Glycosyl transferase, family 2	7057924–7058847
YP_724197	636834370	Tery_4771	Glycosyl transferase, family 2	7329873–7332980
YP_724341	636835285	Tery_4954	Glycosyl transferase, family 2	7547130–7548305

**Table 5:** FAS1-containing genes from *Trichodesmium erythraeum* IMS101, *Anabaena* sp. PCC 7120, and other 15 species.

NCBI Accession	IMG Accession	Gene	Species
NP_485363	4222220	Alr1320 Alr1320 protein	*Anabaena* sp. PCC 7120
NP_485859	4227250	Alr1819 Alr1819 protein	*Anabaena* sp. PCC 7120
NP_487837	4247260	All3797 All3797 protein	*Anabaena* sp. PCC 7120
NP_488687	4255850	All4647 All4647 protein	*Anabaena* sp. PCC 7120
NP_488934	4258350	All4894 All4894 protein	*Anabaena* sp. PCC 7120
NP_489304	4262100	All5264 All5264 protein	*Anabaena* sp. PCC 7120
YP_722947	636827305	Tery_3372 beta-Ig-H3/fasciclin	*Trichodesmium erythraeum* IMS101
YP_722948	636827310	Tery_3373 beta-Ig-H3/fasciclin	*Trichodesmium erythraeum* IMS101
AAF02137	—	Unknown protein	*Arabidopsis thaliana*
CAF32145	—	Fasciclin I family protein, putative	*Aspergillus fumigatus*
EAQ86204	—	Hypothetical protein CHGG_07457	*Chaetomium globosum* CBS 148.51
EAM48409	—	Beta-Ig-H3/fasciclin	*Crocosphaera watsonii* WH 8501
AAW46332	—	Hypothetical protein CNK01730	*Cryptococcus neoformans* var. neoformans JEC21
CAI83309.1	—	Fasciclin domain protein	*Dehalococcoides* sp. CBDB1
EAS19928	—	Putative cell adhesion protein, fasciclin domain	*Flavobacteria* bacterium BBFL7
AAO92753	—	Arabinogalactan protein	*Gossypium hirsutum*
BAC65875	—	Putative membrane-associated or secreted protein	*Magnaporthe grisea*
AAM05399	—	Hypothetical protein MA_1996	*Methanosarcina acetivorans* C2A
ZP_00108174	—	COG2335	*Nostoc punctiforme* PCC 73102
CAH58718	—	Fasciclin-like protein precursor	*Physcomitrella patens*
CAA20163	—	Putative secreted protein	*Streptomyces coelicolor* A3(2)
AAB62187	—	Putative secreted protein MPB70	*Synechococcus* sp. PCC 7002
AAC49869	—	Endosperm specific protein	*Zea mays*
